# Neuromuscular electrical stimulation for preventing skeletal-muscle weakness and wasting in critically ill patients: a systematic review

**DOI:** 10.1186/1741-7015-11-137

**Published:** 2013-05-23

**Authors:** Nicola A Maffiuletti, Marc Roig, Eleftherios Karatzanos, Serafim Nanas

**Affiliations:** 1 Neuromuscular Research Laboratory, Schulthess Clinic, Zurich, Switzerland; 2 School of Physical and Occupational Therapy, McGill University, Montreal, Canada; 3 Department of Exercise and Sport Sciences and Department of Neuroscience and Pharmacology, University of Copenhagen, Copenhagen, Denmark; 4 First Critical Care Department, Evangelismos Hospital, National and Kapodistrian University of Athens, Athens, Greece

**Keywords:** Muscle strength, Muscle mass, Quadriceps femoris, Intensive care, Sepsis, Rehabilitation

## Abstract

**Background:**

Neuromuscular electrical stimulation (NMES) therapy may be useful in early musculoskeletal rehabilitation during acute critical illness. The objective of this systematic review was to evaluate the effectiveness of NMES for preventing skeletal-muscle weakness and wasting in critically ill patients, in comparison with usual care.

**Methods:**

We searched PubMed, CENTRAL, CINAHL, Web of Science, and PEDro to identify randomized controlled trials exploring the effect of NMES in critically ill patients, which had a well-defined NMES protocol, provided outcomes related to skeletal-muscle strength and/or mass, and for which full text was available. Two independent reviewers extracted data on muscle-related outcomes (strength and mass), and participant and intervention characteristics, and assessed the methodological quality of the studies. Owing to the lack of means and standard deviations (SDs) in some studies, as well as the lack of baseline measurements in two studies, it was impossible to conduct a full meta-analysis. When means and SDs were provided, the effect sizes of individual outcomes were calculated, and otherwise, a qualitative analysis was performed.

**Results:**

The search yielded 8 eligible studies involving 172 patients. The methodological quality of the studies was moderate to high. Five studies reported an increase in strength or better preservation of strength with NMES, with one study having a large effect size. Two studies found better preservation of muscle mass with NMES, with small to moderate effect sizes, while no significant benefits were found in two other studies.

**Conclusions:**

NMES added to usual care proved to be more effective than usual care alone for preventing skeletal-muscle weakness in critically ill patients. However, there is inconclusive evidence for its benefit in prevention of muscle wasting.

## Background

A large majority of patients admitted to the intensive care unit (ICU) after the very acute phase of a critical illness exhibit major defects in skeletal-muscle strength (weakness) and mass (wasting) [1-3]. This so-called ICU-acquired weakness (ICUAW) is generally defined as a bilateral deficit of muscle strength in all limbs [4], which is accompanied by a profound loss of muscle mass (as high as 5% per day during the first week of ICU stay [5,6]), and is associated with delayed weaning from mechanical ventilation [7], protracted and costly stays in ICU and hospital stay (the average daily ICU cost being approximately €1,000 [8]), and high mortality rates [9,10]. ICUAW, whose etiology is multi-factorial, is associated with impaired physical function and health status in patients who have spent time in ICU, which can persist even years after hospital discharge [11,12]. This drastically increases the duration of post-ICU treatments (including rehabilitation), and provokes severe social, psychological, and economic consequences (the average cost per life-year gained being approximately €6,000 [8]), thus affecting quality of life and delaying return to physical self-sufficiency and return to work of people who have been critically ill.

Because early rehabilitation/mobilization in the ICU has been shown to enhance short-term and potentially long-term functional outcomes [13-15], the use of physical-therapy strategies to counteract skeletal-muscle weakness and wasting has been promoted frequently in the past few years [16-20]. Neuromuscular electrical stimulation (NMES), a technique that consists of generating visible muscle contractions with portable devices connected to surface electrodes [21], has been shown to be effective in treating impaired muscles [22] as it has the potential to preserve muscle-protein synthesis and prevent muscle atrophy during prolonged periods of immobilization [23]. ICU-based NMES has recently been introduced for the treatment of ICUAW, as it does not require active patient cooperation, has an acute beneficial systemic effect on muscle microcirculation [24], and seems to provide some structural and functional benefits to critically ill patients [25]. However, owing to the heterogeneity of the critically ill patient group and also of the NMES procedures implemented in ICUs [18,26-28], the effectiveness of this rehabilitation procedure for ICUAW prevention remains to be clearly proven.

Previous reviews have analyzed the effect of NMES on different muscle outcomes in patients with specific chronic diseases such as chronic obstructive pulmonary disease (COPD) [22]. Since those reviews were published, several randomized controlled trials (RCTs) have been completed. Furthermore, a detailed analysis of the effects of NMES in critically ill patients is lacking. Results from previous studies suggest that the most deconditioned patients obtain the best results when NMES is applied [22]. Given the potential use of NMES among patients with a limited capacity to engage in voluntary muscle work, assessment of the evidence for the use of NMES in critically ill patients is urgently needed. We therefore undertook a formal systematic review of the literature to determine the rehabilitative effect of NMES on skeletal-muscle strength and mass in critically ill patients, in comparison with standard care.

## Methods

### Electronic search and information sources

Although we developed a review protocol and followed Preferred Reporting Items for Systematic Reviews and Meta-Analyses (PRISMA) guidelines (see Additional file 1) [29], the study protocol was not registered. Two of the authors (EK, SN) independently performed the electronic search on the following databases: PubMed (1951 to present), Cochrane Controlled Trials Register (CENTRAL) (1894 to present), Cumulative Index to Nursing and Allied Health Literature (CINAHL) (1981 to present), Web of Science (1970 to present) and Physiotherapy Evidence Database (PEDro) (1929 to present). Reference lists from articles related to the topic were also searched. The search was not language-restricted but it was limited to RCTs completed on human subjects. The terms used to perform the search were: electrotherapy, electrical stimulation, electrical muscle stimulation, electromyostimulation, electrostimulation, neuromuscular stimulation, and NMES. The results of the primary search were combined with the terms: critically ill patients, critical illness, intensive care, and ICU. For instance, these terms were combined as follows to build the search in PubMed: (electrotherapy OR electrical stimulation OR electrical muscle stimulation OR electromyostimulation OR electrostimulation OR neuromuscular stimulation OR NMES) AND (critically ill patients OR critical illness OR intensive care OR ICU). As additional filters, clinical trial (in ‘Article types’) and humans (in ‘Species’) were chosen. The latest electronic search was performed on March 3, 2012.

### Study selection and eligibility criteria

The list of titles and abstracts of articles retrieved in the electronic search were first reviewed independently by two of the authors (EK, NAM), who selected only those potentially relevant for a more detailed review at full-text level. Both reviewers then read the full text and applied the following inclusion criteria: RCTs 1) exploring the effect of NMES in critically ill patients; 2) with a well-defined NMES protocol (that is, the main stimulation parameters were provided) for at least one intervention group; 3) with NMES applied to skeletal muscles with an intensity equal to or greater than motor threshold (that is, evoking a visible muscle contraction); 4) including outcomes related to muscle strength and/or mass; (5) and whose full text was available. After reviewing the articles and applying the inclusion criteria independently, both reviewers held a consensus meeting to compare their results and decide which articles should finally be included in the review. In cases of disagreement, a third reviewer (MR) was included in the discussion to reach a final consensus.

### Data collection process

Two of the authors (EK, MR) independently extracted the data from the studies included in the review. Data retrieved included characteristics of patients (number, gender, age, diagnosis, and disease severity), interventions (type, duration, frequency, and NMES parameters), and muscle-related outcomes. When provided, details on the number of patients excluded or discharged and their compliance with treatment were also recorded. After extraction, both reviewers compared their data-extraction sheets to confirm the accuracy of the data.

### Methodological quality

Two authors (EK and MR independently) assessed the methodological quality of the studies using the PEDro scale. This scale, which has been used extensively in the methodological evaluation of similar studies [22], and has previously shown good validity and reliability [30,31], is based on 11 items for assessing scientific rigor: eligibility criteria, random allocation, concealed allocation, baseline comparability, blinded subjects, blinded therapists, blinded assessors, follow-up, intention-to-treat (ITT), between-group analysis, both point estimates, and variability. Ten of the items were used in this study to calculate the final score (maximum 10 points). The one item not used was eligibility criteria, which was excluded because it affects external but not internal or statistical validity. We compiled an arbitrary scale of quality, based on PEDro score, with high quality being a score greater than 5, moderate quality being 4 or 5, and low quality being 3 or lower [32]. To minimize errors and potential biases in the methodological evaluation, both reviewers compared their scores in a consensus meeting. In cases of disagreement, a third reviewer (NAM) was included in the discussion to reach a final consensus. Consistency between the two reviewers who performed the methodological assessment (PEDro scores) was evaluated with the Cronbach’s coefficient α. Overall methodological quality based on the PEDro scores was also categorized in accordance with the indications provided by Van Tulder *et al.*[33].

### Data analysis

Outcomes were grouped into two main categories for data analysis: muscle strength and muscle mass (thickness and volume). Probably because data were not normally distributed, the majority of the studies included in the review reported continuous outcomes using medians with interquartile range instead of means with standard deviation (SD). We used several statistical approaches [34] in an attempt to normalize data distribution for the three studies that had raw data available [25,35,36], but we failed to alter the skewed data distribution. We also used the equations proposed by Hozo *et al.* to estimate means and SDs from medians, range, and sample size [37]. However, none of these approaches allowed us to calculate means and SDs reliably. In addition, because of the critical status of some of the study participants, muscle strength was not assessed at admission, and therefore baseline strength measurements were not obtained in two studies [35,36]. Given these two important limitations, it was not possible to pool the data from the different studies to conduct a full meta-analysis. Instead, when means and SDs were provided, we calculated the effect size (*d*) of individual outcomes by dividing the difference between mean change scores (post-intervention minus pre-intervention scores) by the pooled SD [38]. Effect sizes were then categorized in accordance with the criteria established by Cohen as large (*d*> 0.8), moderate (*d*< 0.8 but > 0.2), or small (*d*< 0.2) effects [38]. When means and SDs at baseline and after the intervention were not provided, individual effect sizes were not calculated and, instead, a qualitative analysis of the data was performed.

## Results

### Study selection

**Figure 1:**
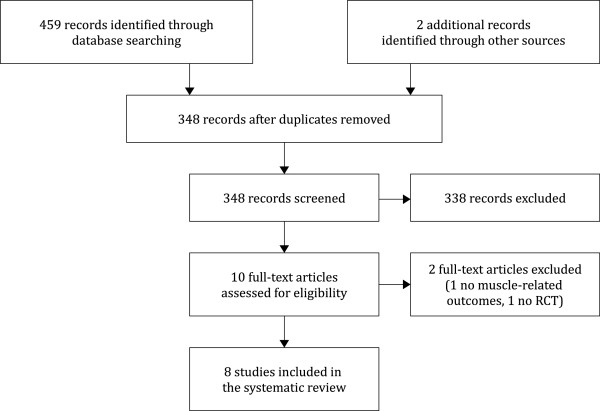
Flow diagram of search strategy.

The different steps of the electronic search are illustrated in Figure 1. The initial search yielded 461 articles, which were included in the review process at abstract level. After 113 duplicates were removed and 348 records were screened, only ten full-text articles could be assessed for eligibility (336 records were excluded because they did not meet all the required inclusion criteria, and two further studies were excluded because they were conference proceeding abstracts and the full text was not available [39,40]). Of those ten articles, two were excluded; one was not an RCT [41], and the other did not report any relevant muscle-related outcome [42]. Finally, the remaining eight RCTs met all the required criteria and were included in the systematic review [25,35,36,43-47]. It should be noted, however, that one of the selected articles [35] presented a secondary analysis of the same study reported in another article [36]; however, because these two studies reported data from different outcomes, we presented them individually [35,36].

### Methodological quality

The PEDro score for each study is reported in Table 1. The mean ± SD PEDro score of the studies included in the review was 5.5 ± 1.5, with scores ranging from 4 to 8 (that is, moderate to high quality). When PEDro scores of the two reviewers were compared, consistency was high (α=0.751; *P*<0.0001) [48]. The most common methodological weaknesses of the studies referred to the blinding of patients (although sham NMES was used in two studies [43,44], which could be considered a type of blinding), therapists, and assessors. The allocation of subjects to different intervention groups was concealed in only two studies [43,46]. In addition, two studies did not report baseline data for muscle strength, and therefore comparability between groups could not be established [35,36]. Only three studies met the follow-up criteria as established by the PEDro scale [43,45,46], either because data for at least one key outcome were not obtained in more than 15% of the patients initially allocated into treatment groups [25,35,36], or because the number of patients from whom key outcome data were obtained was not explicitly stated [44,47]. Two of the studies used ITT analysis [35,36], and in one study all patients received treatments as allocated [46]. The rest of the studies did not meet the ITT analysis criterion [25,43-45,47]. The results of all five RCTs that investigated the effect of NMES on muscle strength supported the effectiveness of this intervention. Because two of these studies [43,46] were of high methodological quality (PEDro score ≥ 7), the level of evidence could be categorized as moderate to strong. By contrast, because only two [25,44] of the four studies that investigated the effect of NMES on muscle mass found a positive outcome, the evidence in support of this technique to improve muscle mass can be considered as conflicting.

**Table 1:** Methodological quality of the studies included in the systematic review (PEDro scores)

	**Abdellaoui *et al. *2011 **[42]	**Gerovasili *et al*. 2009 **[25]	**Gruther *et al. *2010 **[43]	**Karatzanos *et al. *2012 **[34]	**Poulsen *et al. *2011 **[44]	**Rodríguez *et al. *2011 **[45]	**Routsi *et al. *2010 **[35]	**Zanotti *et al. *2003 **[46]
Random allocation	✓	✓	✓	✓	✓	✓	✓	✓
Concealed allocation	✓					✓		
Baseline comparability	✓	✓	✓		✓	✓		✓
Blinded subjects	✓		✓					
Blinded therapists								
Blinded assessors		✓	✓		✓	✓		
Follow-up	✓				✓	✓		
Intention-to-treat				✓		✓	✓	
Between-group analysis	✓	✓	✓	✓	✓	✓	✓	✓
Point estimates and variability	✓	✓	✓	✓	✓	✓	✓	✓
Total score	7/10	5/10	6/10	4/10	6/10	8/10	4/10	4/10

PEDro, Physiotherapy Evidence Database.

### Participants

Characteristics of the patients included in the review are shown in Table 2. We included in the review (at the study level) only that information on patients for whom data for at least one of the outcomes of interest was provided. Data from 172 patients (46 female, 126 male) were retrieved. Of those 172 patients, 74 were allocated to the NMES group and 76 to the control group, while the remaining 22 patients received NMES on one side of the body and the contralateral side acted as control. The most common diagnoses at admission were sepsis, COPD, and trauma, although patients were also hospitalized because of neurological problems, cancer, or post-surgery complications. The severity of the disease was categorized by the Simplified Acute Physiology Score III (SAPS III) [25,35,36], the Sequential Organ Failure Assessment (SOFA), and the Acute Physiology and Chronic Health Evaluation II (APACHE II) [25,35,36,45,46]. No severity scores were reported in one study [44]. In addition, two studies reported the number of patients diagnosed with critical illness polyneuromyopathy [35,36], and three other studies reported the number of days in the ICU [45-47]. Two studies that investigated the effects of NMES on patients with COPD reported spirometry and blood gas values as measures of disease severity [43,47]. According to international guidelines [49], those patients would be categorized as patients with severe to very severe COPD.

**Table 2:** Characteristics of the patients included in the systematic review

**Study**	**Sample size**^**a**^**(% men)**	**Age, years**^**b**^	**Diagnoses**	**Disease severity**^**b**^			
				**SAPS III**	**SOFA**	**APACHE III**	**Other**
Abdellaoui *et al.*[42]	C: 6 (100%)	C: 67(59–72)	COPD				C: FEV_1_ of 15(10–27)%
	N: 9 (78%)	N: 59(57–69)					N: FEV_1_ of 25(17–41)%
Gerovasili *et al.*[25]	C: 13 (62%)	C: 56(19)	Sepsis	C: 61(14)	C: 8(3)	C: 18(6)	
	N: 13 (46%)	N: 59(23)	Trauma	N: 66(9)	N: 10(3)	N: 19(3)	
			Neurologic				
Gruther *et al.*[43]	C(A): 9 (89%)	C(A): 48(12)	Polytrauma				
	N(A): 8 (88%)	N(A): 52(10)	Cardiovascular				
	C(L): 8 (50%)	C(L): 64(8)	Transplant				
	N(L): 8 (88%)	N(L): 61(10)	Pneumonia				
			Cancer				
Karatzanos *et al.*[34] and Routsi *et al.*[35]	C: 28 (79%)	C: 59(21)	Sepsis	C: 58(14)	C: 8(3)	C: 19(5)	C: 39% with CIPNM
	N: 24 (79%)	N: 55(20)	Trauma	N: 55(11)	N: 8(3)	N: 16(4)	N: 13% with CIPNM
			Post-surgery				
			Brain injury				
			Respiratory failure				
Poulsen *et al.*[44]	C/N: 8 (100%)	C/N: 67(64–72)	Sepsis		C/N: 11(9–14)	C/N: 25(20–29)	C/N: 13(10–22) days in ICU
Rodríguez *et al.*[45]^*c*^	C/N: 14 (50%)	C/N: 72(63–80)	Sepsis		C/N: 10(9–12)	C/N: 20(18–27)	C/N: 27(19–44) days in ICU
Zanotti *et al.*[46]	C: 12 (67%)	C: 65(4)	COPD				C: 47(19) days in ICU
	N: 12 (75%)	N: 66(8)					N: 52(15) days in ICU

A, acute; APACHE III, Acute Physiology and Chronic Health Evaluation III; C, control group; CIPNM, critical illness polyneuromyopathy; COPD, chronic obstructive pulmonary disease; FEV_1_, forced expiratory volume in 1 second; ICU, intensive care unit; L, long-term; N, neuromuscular electrical stimulation group; SAPS III, Simplified Acute Physiology Score III; SOFA, Sequential Organ Failure Assessment.

^a^Only patients included in the analysis of the outcomes of interest are shown.

^b^Data are provided as means (standard deviation) or medians (interquartile range).

^c^The contralateral side acted as control.

### Interventions

The characteristics of the interventions are shown in Table 3. The duration of the NMES protocol ranged from 7 days to 6 weeks. Patients in the control group received usual care and, in some studies, either assisted limb mobilization with [43] or without sham NMES [47], or sham NMES alone [44]. NMES was delivered while the patient was relaxed, and targeted the following muscle groups: glutei [47], quadriceps [25,35,36,43-47], hamstrings [43], peroneus longus [25,35,36], and biceps brachii [46]. Specific details of the stimulation parameters are shown in Table 3. In all studies, the criterion to establish the minimum intensity of NMES was a visible muscle contraction, which corresponds to the motor threshold [50]. NMES intensity during the treatment was progressively adjusted to the individual patient’s tolerance or set as a percentage (150%) of the motor threshold [45]. Stimulation frequencies ranged from 8 to 100 Hz, and pulse durations from 250 to 400 μs. Five studies reported the use of symmetric biphasic pulses [25,35,36,43,46], and one reported the use of asymmetric currents [47]. The shape (rectangular) of the stimulation pulse and the ramp-up and ramp-down times were reported in only three studies [35,36,45]. In general, compliance (percentage of sessions completed) with NMES treatment was high (81 to 100%), but compliance was not reported in two studies [44,47]. No adverse events or complications in relation to NMES safety or tolerability were reported in seven of the eight studies included in the systematic review [45]. For the remaining study, superficial skin burns and excessive pain occurred in one and two patients, respectively, out of fourteen patients treated by Rodriguez *et al.*[46].

**Table 3:** Intervention characteristics, outcomes and main results of the studies included in the systematic review

**Study**	**Interventions by group/side**		**NMES parameters**	**Outcomes**^**a **^**(tools)**	**Main results**
	**C**	**N**			
Abdellaoui *et al.*[42]	ALM + sham NMES to quadriceps and hamstrings	ALM + NMES to quadriceps and hamstrings (BL): 60 min/day × 5 days/week × 6 weeks	Frequency: 35 Hz	Muscle strength (dynamometry)	Quadriceps strength increased more for N than C (*p* < 0.01)
			Pulse duration: 400 μs		
			Intensity: 15-32 mA for quadriceps, 22-47 mA for hamstrings (start-end)		
Gerovasili *et al.*[25]	Usual care	Usual care + NMES to quadriceps and peroneus longus (BL): 55 min/day × 8 days	Frequency: 45 Hz	Muscle thickness (US)	Rectus femoris and vastus intermedius (right side) thickness decreased less for N than C (*p* < 0.05); *d* = 0.11-0.39 (small-moderate)
			Pulse duration: 400 μs		
			On-off ratio: 12-6 s		
			Intensity: 37-38 mA (mean)		
Gruther *et al.*[43]	Sham NMES	NMES to quadriceps (BL): 30-60 min/day × 5 days/week × 4 weeks	Frequency: 50 Hz	Muscle thickness (US)	Quadriceps thickness increased only for N (long-term patients) (*p* < 0.13); *d* = 0.36 (moderate)
			Pulse duration: 350 μs		
			On-off ratio: 8-24 s		
			Intensity: tolerance		
Karatzanos *et al.*[34]	Usual care	Usual care + NMES to quadriceps and peroneus longus (BL): 55 min/day × 7 days/week until ICU discharge	Frequency: 45 Hz	Muscle strength (MRC)	MRC scores for wrist flexion, hip flexion, ankle dorsiflexion (*p* < 0.05) and knee extension (*p* < 0.01) were greater for N than C
			Pulse duration: 400 μs		
			On-off ratio: 12-6 s		
			Intensity: motor threshold		
Poulsen *et al.*[44]	Contralateral side acted as control	NMES to quadriceps (UL): 60 min/day × 7 days	Frequency: 35 Hz	Muscle volume (CT)	Quadriceps volume decreased for both C and N, with no difference between sides (*p* = 0.1)
			Pulse duration: 300 μs		
			On-off ratio: 4-6 s		
			Intensity: motor threshold		
			+50% (adjusted daily)		
Rodríguez *et al.*[45]	Contralateral side acted as control	NMES to biceps brachii and quadriceps (UL): 2 × 30 min/day × 13 days	Frequency: 100 Hz	Muscle strength (MRC)	MRC scores for elbow flexion (*p* = 0.005) and knee extension (*p* = 0.034) were greater for N than C. Biceps thickness was unchanged
			Pulse duration: 300 μs		
			On-off ratio: 2-4 s	Muscle thickness (US)	
			Voltage: 20-200 V		
Routsi *et al.*[35]	Usual care	Usual care + NMES to quadriceps and peroneus longus (BL): 55 min/day × 7 days/week until ICU discharge	Frequency: 45 Hz	Muscle strength (MRC)	Global MRC score was greater for N than C (*p* = 0.04)
			Pulse duration: 400 μs		
			On-off ratio: 12-6 s		
			Intensity: motor threshold		
Zanotti *et al.*[46]	ALM: 5 days/week × 4 weeks	ALM + NMES to quadriceps and glutei (BL): 25-30 min/day × 5 days/week × 4 weeks	Frequency: 8-35 Hz	Muscle strength (MRC)	MRC score increased more for N than C (*p* < 0.02); *d* = 1.44 (large)
			Pulse duration: 250-350 μs		
			Intensity: motor threshold		

ALM, active limb mobilization; BL, bilateral; C, control group; MRC, Medical Research Council; N, NMES group; NMES, neuromuscular electrical stimulation; US, ultrasonography.

### Outcomes

#### Muscle strength

Five studies assessed the effects of NMES on strength of different muscle groups (Table 3) [35,36,43,46,47]. Four studies evaluated muscle strength using the Medical Research Council (MRC) scale [35,36,46,47]. One study found a significantly larger MRC score increase in the NMES group compared with the control group [47], with a large effect size (*d* = 1.44). Two studies reported greater MRC scores in the NMES group than in the control group [35,36], although baseline measurements were not provided. Another study found significantly higher MRC scores on the stimulated side compared with the contralateral side [46]. One study, in which quadriceps muscle strength was assessed by dynamometry [43], reported a significantly larger strength increase in the NMES group compared with the control group.

#### Muscle mass

Four studies assessed the effects of NMES on muscle thickness [25,44,46], or volume [45] (Table 3). Muscle thickness was measured with ultrasonography, and muscle volume was obtained from the analysis of computed tomography images. In one study, muscle thickness decreased less in the NMES group than in the control group [25], and effect sizes (*d*) ranged from 0.11 to 0.39, depending on the muscle group assessed. Another study investigated the effects of NMES on quadriceps muscle thickness in acute (less than 7 days hospitalization) and long-term (greater than 14 days hospitalization) patients, and found that thickness increased only for long-term patients (*d* = 0.36) but not for acute or sham patients [44]. The two other studies found no significant changes in muscle thickness between the stimulated and contralateral biceps brachii [46], and no differences in muscle volume loss between the stimulated and contralateral quadriceps [45].

## Discussion

Neuromuscular electrical stimulation added to usual care, in comparison with usual care alone or sham stimulation, was associated with better muscle-strength outcomes in patients in the ICU, with moderate to strong evidence. However, the level of evidence was weaker and conflicting for outcomes related to muscle mass, with small to moderate effect sizes or no effect. These findings suggest that NMES may have the potential to prevent skeletal-muscle weakness in critically ill patients, which could confer many important physical, psychosocial, and economic benefits for these patients after discharge from ICU. However, it remains to be ascertained whether NMES therapy can also prevent the muscle wasting associated with critical illness.

The high inconsistency in ICU patient characteristics between studies was not unexpected (as attested by non-normal data distribution and lack of means and SDs), but it affected the methodological quality of the included studies, which prevented us from completing a meta-analysis. Therefore, the main results of this systematic review could only be interpreted with a thorough qualitative analysis. Although it is extremely challenging to perform large and well-controlled RCTs in this patient population, future NMES studies should consider stratifying patients for main diagnosis and eventually also for disease severity, as this latter feature has been identified as an independent risk factor for ICUAW incidence [10,51]. It is conceivable that the benefits of NMES are greater for patients admitted to the ICU with respiratory complications (as suggested by the large effect sizes for patients with COPD) [47], or neurological complications, compared with patients with sepsis or trauma. For example, inflammation-mediated electrolyte changes and also edema may seriously affect conductivity and thus electrical current diffusion [52], which could lessen any systemic effect of NMES in these patient samples.

The questionable validity and heterogeneity of the NMES protocol characteristics adopted in the eight studies included in this systematic review further complicated the interpretation of the present results. The strength of the contraction induced by NMES (that is, evoked tension), which is the main determinant of NMES effectiveness [53], was not reported in any of the included studies. Quantifying this parameter, rather than stating simple current intensity/voltage, is crucial as it would also permit discrimination of responders from non-responders [54,55], and eventually allows ascertainment of the optimal NMES characteristics for patients in the ICU on an individual basis. In addition, evoked tension should be maximized, whenever possible, by selecting appropriate current parameters (stimulation frequency of 50 to 100 Hz [56] and highest tolerable stimulation intensity, while minimizing fatigue with long relaxation phases), joint position (long muscle length), and methodological precautions such as the accurate determination of muscle motor points [57].

Assessing voluntary muscle strength in the ICU is extremely difficult. Despite potential limitations of manual muscle testing such as poor validity and inaccuracy of subjective ratings [58,59], especially when assessors are not blinded, evaluation of voluntary strength using the MRC score was used in the majority of the included RCTs, and only one study used dynamometry [43]. Considering the limited or absent cooperation of patients at admission into the ICU, and the considerable influence of central factors (including motivation) on maximal voluntary efforts [60], it would be preferable if evaluation of muscle function in these patients relied on artificially-evoked muscle responses. Therefore, alternative methods that are independent of patient cooperation such as peripheral magnetic stimulation (which can also be used to evaluate respiratory muscle function) [61], electrical impedance myography [62], myotonometry [63], and mechanomyography [64], would improve the validity of muscle testing in ICU.

The major risk factors for ICUAW are immobilization, multiple organ failure, systemic inflammatory response syndrome, gram-negative septicemia [10], hyperglycemia [3,65], and medications such as aminoglycosides, colistin, and corticosteroids. All these elements should be viewed as important confounding factors that might distort accurate interpretations of our findings. For example, patients with a recent exacerbation of COPD (most of whom are prescribed corticosteroids, which can induce myopathy) were excluded in one instance [47], whereas another study examined the effects of NMES after COPD exacerbation (some patients received corticosteroids) [43]. Studies must always carefully control for immobilization days, disease severity scores (organ specific and physiological), and medication use.

Even though physical-therapy practices vary widely between different ICUs, there is growing interest in early rehabilitation strategies that have the potential to prevent skeletal-muscle weakness and wasting in critically ill patients [20]. These interventions range from passive stretching [5] and early mobilization therapy [3] to bedside cycling ergometry [13]. Interestingly, NMES added to usual care has recently been shown to be effective in reducing ICUAW incidence [36]. The present systematic review confirms these preliminary findings, highlighting the potential role of NMES as a preventive countermeasure against ICUAW. Compared with other rehabilitation strategies, the unique aspects of NMES are that it is relatively cost-effective (one multiple-user NMES unit costs less than €400), does not require patient cooperation (it can be applied to sedated patients) or stable cardiac or respiratory function, can be implemented during the first few days after ICU admission, and provokes considerable central effects, both acute and chronic [66], which could also contribute to preventing the occurrence of muscle weakness in critically ill patients. Moreover, in addition to muscle-related outcomes, NMES has been shown to be more effective than conventional care or sham stimulation for improving pulmonary function [36,43,47], including accelerated weaning from mechanical ventilation [36], physical function (6-minute walking distance [43] and bed to chair transfer [47]), and for reducing the incidence of critical illness polyneuromyopathy [36]. However, the effects of NMES on the pathophysiological mechanisms of ICUAW are poorly known, and NMES cannot be easily used with all critically ill patients (for example, those with skin lesions, traumatic fractures, complete lower motor-neuron lesions and cardiac pacemakers), so that there is still no consensus among intensive care specialists about its real value.

### Limitations

The major limitation of the present review concerns the unavailability of outcome data (for example, baseline strength measurements) to allow a full meta-analysis to be conducted. At face value, this lack of data could be indicative of reporting bias at outcome level. However, rather than reporting bias, lack of outcome data should simply be seen as one of the many limitations inherent in studies conducted on patients admitted to the ICU. We factorized potential biases at study level by assessing the methodological quality of the studies, which allowed us to assess the reliability and validity of the data and to weigh the results of each study based on its methodological rigor. Unfortunately, because of the impossibility of calculating the effect sizes in many of the studies included in the review, the risk of publication bias could not be assessed.

## Conclusions

This systematic review provides evidence that adding NMES therapy to usual care is more effective than usual care alone or sham NMES in preventing ICUAW. Nevertheless, there is inconclusive evidence about the effectiveness of NMES for the preservation of muscle mass in ICU patients. The effects of NMES we found were probably underestimated because of the non-stratification of patients according to main diagnosis and disease severity. More studies are needed to explore the long-term effects of NMES therapy during ICU stay on physical function and quality of life in ICU survivors, in order to identify the optimal NMES dosage for ICUAW prevention (both in terms of frequency, intensity and volume), and to describe the feasibility, safety, and cost-effectiveness of NMES in different subpopulations of critically ill patients.

## Abbreviations

APACHE: Acute physiology and chronic health evaluation; CENTRAL: Cochrane controlled trials register; CINAHL: Cumulative index to nursing and allied health literature; COPD: Chronic obstructive pulmonary disease; ICU: Intensive care unit; ICUAW: Intensive care unit acquired weakness; ITT: Intention-to-treat; MRC: Medical research council; NMES: Neuromuscular electrical stimulation; PEDro: Physiotherapy evidence database; PRISMA: Preferred reporting items for systematic reviews and meta-analyses; RCT: Randomized controlled trial; SAPS: Simplified Acute Physiology Score; SD: Standard deviation; SOFA: Sequential organ failure assessment.

## Competing interests

The authors declare that they have no competing interests.

## Authors’ contributions

NAM and MR made substantial contribution to conception and design of the review. All authors made substantial contribution to data acquisition, analysis, and interpretation. All authors were involved in drafting and critically revising the manuscript. All authors approved the final manuscript.

## Pre-publication history

The pre-publication history for this paper can be accessed here:

http://www.biomedcentral.com/1741-7015/11/137/prepub

## Supplementary Material

Additional file 1 PRISMA 2009 checklist. Click here for file
